# Protective effect of chicken yolk antibody Y against *Campylobacter jejuni* induced diarrhea in cats

**DOI:** 10.3389/fmicb.2024.1378029

**Published:** 2024-04-09

**Authors:** Ziyang Li, Jiayi Yan, Zhaowei Bian, Jie Zhang, Yuwen Liu, Jinping Deng, Baichuan Deng, Sufang Han

**Affiliations:** Guangdong Provincial Key Laboratory of Animal Nutrition Control, College of Animal Science, South China Agricultural University, Guangzhou, China

**Keywords:** *Campylobacter jejuni*, IgY, inflammation, metabolism, cats

## Abstract

*Campylobacter jejuni (C. jejuni)* is a common pathogen that often causes diarrhea, loss of appetite, and even enteritis in domestic cats, affecting their growth and development, especially in kittens under 6 months of age. Oral passive immunization with chicken yolk antibody Y has been proved effective for the treatment of gastrointestinal pathogen infections due to its high specificity. In this study, *C. jejuni* was isolated from diarrheal cat feces, and the specific egg yolk antibody Y against *C. jejuni* was demonstrated to effectively inhibit its proliferation *in vitro* experiments. To evaluate the effect of anti-*C. jejuni* IgY, the mouse *C. jejuni* infection model was established and it was found that IgY could alleviate *C. jejuni*-induced clinical symptoms. Consistent with these results, the reduction of pro-inflammatory factors and intestinal colonization by *C. jejuni* in the IgY-treated groups, especially in the high dose group. We then evaluated the protective effect of IgY on young Ragdoll cats infected with *C. jejuni*. This specific antibody reduced the rate of feline diarrhea, protected the growth of young cats, inhibited systemic inflammatory hyperactivation, and increased fecal short-chain fatty acid concentrations. Notably, IgY may have a protective role by changing intestinal amino acid metabolism and affecting *C. jejuni* chemotaxis. Collectively, specific IgY is a promising therapeutic strategy for *C. jejuni*-induced cat diarrhea.

## 1 Introduction

Pets provide physical and mental health benefits, emotional support, and increased social interaction for their owners (Mcnicholas et al., 2005). In urban areas of China, the pet market has been steadily growing in recent years, with more than 100 million cats and dogs. By 2022, the number of pet cats has surpassed the number of pet dogs.^1^

Bacterial infections can cause diarrhea, especially in multi-cat households (German et al., 2015). Diarrhea can interfere with the intestinal absorption of nutrients, and recurrent episodes can lead to developmental delays in young animals (Stern et al., 1980). At the same time, diarrhea facilitates the spread of enteric pathogens into the environment, which can infect new hosts, and host transmission can increase the virulence of certain bacteria (Wiles et al., 2005; Butler et al., 2006). *Campylobacter jejuni (C. jejuni)* is a potential threat to diarrhea in kittens. Many researchers have found a high prevalence of *C. jejuni* in cats (Gruffyddjones, 1980; Acke et al., 2009; Bojanić et al., 2017; Torkan et al., 2018). Infection with *C. jejuni* can lead to chronic diarrhea and even enteritis in cats (Fleming, 1983). Although the infected cats may not show serious clinical signs, they can still act as a reservoir of *C. jejuni* and infect other cats and humans.

However, there are no very effective strategies for dealing with *C. jejuni* infections other than antibiotics. By collecting fecal samples from pets to isolate *C. jejuni* and perform drug susceptibility testing, it has been discovered that pets harbor *C. jejuni* strains that possess resistance genes for several antibiotics, including gentamicin, kanamycin, streptomycin, and tetracycline (Montgomery et al., 2018; Joseph et al., 2020; Moser et al., 2020; Francois Watkins et al., 2021). The unregulated use of antibiotics can contribute to the development of superbugs that can be transmitted from pets to their owners, causing more difficult-to-treat infections and threatening human health. Therefore, it is important to explore new treatment options for human and pet health.

Passive immunity refers to the specific immunity acquired by the body passively receiving antibodies, sensitizing lymphocytes or their products (Brambell, 2010; Kovacs-Nolan and Mine, 2012). It is a more attractive approach because of its rapid delivery and immediate protection. Eggs are a rich source of nutrients and contain large amounts of antibodies. More importantly, chickens are natural hosts for *C. jejuni*. It is noteworthy that *C. jejuni* is not easily detectable in chicks up to 3 weeks of age, which may be related to antibodies in the egg yolk (Kim et al., 2000). This protective effect probably is likely a result of the passive immune effect of the antibodies provided by the hen through the egg yolk. Three types of antibodies are found in chickens: IgY, IgA, and IgM. During yolk formation, IgY is transferred from serum to yolk, providing passive immunity to chicks (Gerrie et al., 1973; Kovacs-Nolan and Mine, 2012). Since its discovery, IgY has been widely used in diagnostics (Grzywa et al., 2013; Bentes et al., 2015; Jinxin He et al., 2015) and food safety testing (Li et al., 2017; Dou et al., 2022). One of its most valuable and promising areas is its use in passive immunization for the prevention and treatment of human and animal diseases (Pereira et al., 2019; Grzywa et al., 2023). IgY is highly effective against pathogenic infections such as *Salmonella* (Li et al., 2016), *E. coli* (Ma et al., 2020), and *Norovirus* (Dai et al., 2013). In addition, IgY is advantageous due to its high yield (Wang et al., 2023), short preparation cycle, high affinity, and relatively humane preparation process (Karthikeyan et al., 2022). However, there have been few studies investigating the therapeutic and prophylactic effects of IgY in cats infected with *C. jejuni*.

In this study, we isolated a strain of *C. jejuni* from the feces of cats with diarrhea, inactivated it as an antigen, and immunized hens to obtain specific antibodies. We established the mouse *C. jejuni*-induced diarrhea model and found the specific IgY could alleviate *C. jejuni*-induced clinical symptoms. And we demonstrated its protective effect on young Ragdoll cats with *C. jejuni*-induced diarrhea. These findings suggest that the specific IgY is a promising therapeutic strategy for *C. jejuni*-induced diarrhea in cats.

## 2 Materials and methods

### 2.1 Preparation of IgY

Fecal samples were collected from cases of feline diarrhea and used to isolate a strain of *C. jejuni*, which was then identified by biochemical assays and 16S ribosomal DNA sequencing. Bacteria were cultured on Columbia blood agar containing 5% sterile sheep blood and maintained at 42°C under microaerophilic conditions. Anti-*C. jejuni* IgY was produced and purified by Carmel Biotechnology Co Ltd. (Tianjin, China) following the methods previously described by Thibodeau et al. (2017).

### 2.2 Growth inhibition test

The *C. jejuni* (1.0 × 10^5^ CFU/mL) was grown in Mueller-Hinton Broth including a range of density (2.5, 5, and 10 mg/mL) of sterilized anti-*C. jejuni* IgY. Nonspecific IgY (10 mg/mL) (Beyotime Biotechnology Co., Ltd., Shanghai) was performed as the negative control. These mixtures were incubated under microaerobic conditions. Absorbance values were read at 600 nm every 2 h with three replicates. The growth inhibition curve was generated by plotting OD_600_ against time.

### 2.3 Mice and cats infection model

#### 2.3.1 Mice infection model

Thirty-two pathogen-free male C57BL/6 J mice (3 weeks of age, 10.38 ± 0.28 g) were acquired from Spearfish (Beijing) Biotechnology Co. Mice were housed in a controlled room with the same humidity and temperature with a 12 h light–dark cycle. All experimental procedures were authorized by the Animal Care and Use Committee before animal experiments and were performed according to the guidelines of the Laboratory Animal Center of South China Agricultural University (Approval number: 2022F233).

Weaned 3 weeks-old C57BL/6 J were acclimatized on a standard diet for 3 days. The *C. jejuni* infection model was established according to Natasa Giallourou et al. (Baumler et al., 2018). Briefly, before infection, four antibiotics were added to the drinking water to scavenge the intestinal flora and overcome the colonization resistance, and were also fed zinc-deficient diets (zinc content less than or equal to 1 ppm) for 14 days, which were purchased from Nantong Trofi Feeds Science and Technology Co Ltd. (Jiangsu). Mice were given antibiotic-free water and fasted for 4 h the day before infection. Each infected mouse was inoculated with approximately 1 × 10^9^ CFU *C. jejuni* by oral gavage of 100 μL of freshly prepared broth for 3 consecutive days. After the challenge, all mice were randomly divided into four groups according to receive different treatments for 5 days: the control (CON) group received 100 μL of sterile PBS buffer by gavage (*n* = 8), high-dose (HD) group received 10 mg/mL specific IgY (*n* = 8), middle-does group (MD) received 5 mg/mL specific IgY (*n* = 8), and low-dose (LD) group received 2.5 mg/mL specific IgY (*n* = 8). We applied a standardized clinical scoring system to the mice to evaluate the appearance of the mice and the consistency of the feces maximum 12 points, addressing the occurrence of blood in feces (0 points: no blood; 2 points: microscopic detection of blood; 4 points: overt blood visible), diarrhea (0: formed feces; 2: pasty feces; 4: liquid feces), and the clinical aspect (0: normal; 2: ruffled fur, less locomotion; 4: isolation, severely compromised locomotion, pre-final aspect) (Heimesaat et al., 2014).

#### 2.3.2 Cats infection model

Twelve Ragdoll cats, half male and half female (4 months old, 3.475 ± 0.16 kg), were purchased from the Ramical Dogs and Cats Scientific Experimental Base (Guangzhou). Cats were housed in a negative pressure laboratory at a temperature of 26°C. Each cat was housed individually in a cage (108 cm*70 cm*76 cm) to avoid cross-infection and was cleaned daily to maintain cleanliness. All cats were vaccinated and dewormed one month prior to the experiment. No antibiotics or other medications were administered that might interfere with the results. During the acclimatization period, the cats had access to fresh food and drink clean water. All procedures were authorized by the Animal Care and Use Committee and were carried out according to the guidelines of the Laboratory Animal Centre of South China Agricultural University (Approval number: 2021a030).

After a 7 days acclimatization period, 4 months-old Ragdoll cats were divided into two groups according to sex and weight. The presence of *C. jejuni* was excluded before the experiment by collecting fresh fecal samples from the Ragdoll cats and extracting total bacterial DNA. Commercial cat food and filtered water were provided during the experiment. Fasting was enforced for 12 h before the infection. Each cat participant received an oral instillation of 1 mL of freshly prepared bacterial solution containing approximately 1 × 10^10^ CFU *C. jejuni* for 5 consecutive days. Subsequently, cats in the C.j group were orally instilled with 1 mL of sterile PBS daily, and cats in the C. j + IgY group received a daily instilled of 1 mL of 25 mg/mL specific IgY for 7 consecutive days. The dose of specific IgY for administration to Ragdoll cats was determined based on dose conversion methods for different animals (Saadh et al., 2020).

The fecal samples were scored on a 1 to 5 scale (1-liquid diarrhea to 5-dry hard pellets) (Carciofi et al., 2008). A score of 3.5 to 4.0 was considered ideal.

### 2.4 Blood collection and analysis

Blood was collected on days 0 (end of acclimation period), 5 (after modeling), and 13 (end of experiment). Cats were fasted overnight and 3 mL of blood was collected from each cat, allowed to stand for 30 min, centrifuged, and the supernatant transferred to a centrifuge tube and stored at −80°C for further analysis.

Serum albumin (ALB), total protein (TP), globulin (GLOB), and albumin/globulin ratios (A/G) were measured using a commercial kit on an automated blood biochemistry analyzer (Chemray 800, Shenzhen Redu Life Technology, Shenzhen, China). Myeloperoxidase (MPO), procalcitonin (PCT), C-reactive protein (CRP), interleukin-6 (IL-6), interferon-γ (IFN-γ), tumor necrosis factor (TNF-α) and interleukin-1β (IL-1β) were assessed using a commercial feline ELISA kits (MEIMIAN, Jiangsu Meimian Industrial Co., Ltd., Jiangsu, China). Nitric oxide (NO) concentration was measured by the commercial kits (Solarbio, Solarbio Biotechnology Co., Ltd., Beijing, China).

### 2.5 Collection and analysis of fresh feces and cecal contents

#### 2.5.1 Short chain fatty acids

At the end of the experiment, fresh feces from Ragdoll cats and cecal contents from mice were collected to extract SCFAs, which were quantified by gas chromatography–mass spectrometry (GC-MS; Shimadzu, Tokyo, Japan) according to the method described in our previous work (Kang Yang and Jian, 2021).

#### 2.5.2 Relative loads of *C. jejuni* in cecum contents

The relative load of *C. jejuni* in the samples was determined using qPCR by extracting total bacterial DNA from mouse cecal contents and the bacterial universal primer was used as an endogenous reference. The primers are listed in Table 1.

**Table 1:** Primer sequences for qPCR.

Gene name	Forward sequence	Reverse sequence
IL-6	ATAGTCCTTCCTACCCCAATTTCC	CTGACCACAGTGAGGAATGTCCAC
IL-1β	GAAATGCCACCTTTTGACAGTG	TGGATGCTCTCATCAGGACAG
β-actin	CTGTCCCTGTATGCCTCTG	ATGTCACGCACGATTTCC
C.jejuni	TCCATCATATCTTGGGCGCT	AATTTGCTTTGAAAGCATTT
Universal bacterial	GTGSTGCAYGGYYGTCGTCA	ACGTCRTCCMCNCCTTCCTC

### 2.6 Intestinal inflammatory factors

The relative mRNA expression of IL-6 and IL-1β in mouse jejunum and colon was examined by qPCR. The relative expression of genes was determined by 2^−ΔΔCt^ and the β-actin gene was used as an endogenous reference. Primers are listed in Table 1. Metalloproteinase-9 (MMP-9) was analyzed in mouse cecum using ELISA kits (MEIMIAN, Jiangsu Meimian Industrial Co., Ltd., Jiangsu, China).

### 2.7 Fecal untargeted metabolomics analysis

Pretreatment of fresh fecal samples from cats as described in our previous work (Jian et al., 2022). The Thermo Fisher Scientific UPLC-Orbitrap-MS/MS system (Q-Exactive Focus, United States) was used for untargeted metabolomic analysis of fecal samples. Raw data were processed using Compound Discoverer 3.3 software by comparing the mzCloud and mzValut online databases to identify metabolites. Orthogonal Partial Least Squares Discriminant Analysis (OPLS-DA) was performed using SIMCA-P14.1 software. KEGG pathway analysis of differential metabolites was performed using MetaboAnalyst 5.0.

### 2.8 Statistical analysis

All data were analyzed using SPSS 26.0 and graphs were generated using GraphPad Prism 8.0 software. *p* values were determined by unpaired Student’s *t*-test. Significant differences and tendencies were indicated by *p* < 0.05 and *p* < 0.10, respectively. The OPLS-DA model was used to calculate variable importance in the projection (VIP) values. Metabolites with VIP > 1 and *p* < 0.05 were considered differential metabolites. These differential metabolites were functionally annotated using the KEGG database and further mapped to the KEGG pathway database using MetaboAnalyst 5.0.

## 3 Results

### 3.1 Specific IgY inhibited the proliferation of *C. jejuni*

**Figure 1:**
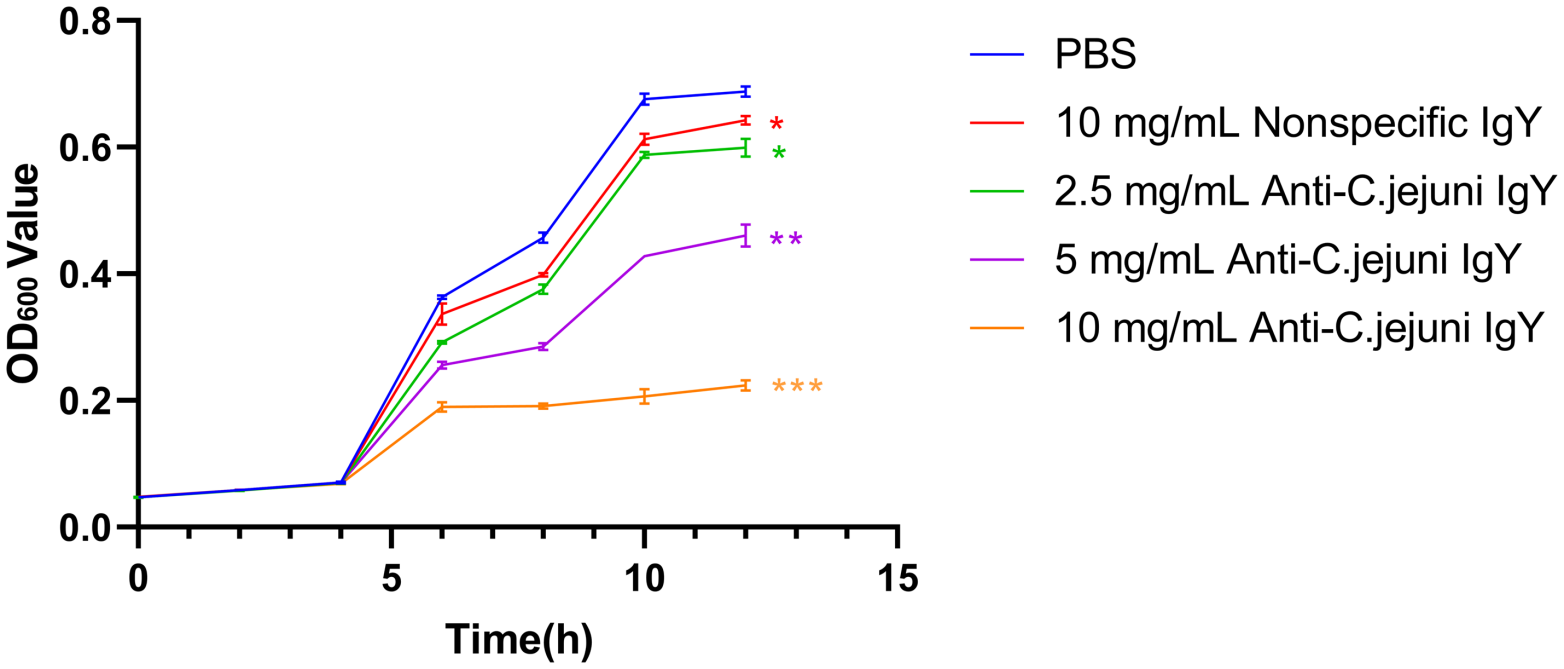
Growth inhibition curves of *C. jejuni* treated with different doses of anti-*C. jejuni* IgY. Values are expressed as the mean ± SEM (*n* = 8). **p < 0.01 and ***p < 0.001 compared to PBS treatment.

To assess the bacteriostatic activity of specific IgY *in vitro*, growth inhibition curves were plotted (Figure 1). The results showed that the anti-*C. jejuni* IgY suppressed the growth of *C. jejuni* in a dose-dependent manner, with the inhibitory effect increasing as the concentrations of IgY increased.

### 3.2 Protective effects of IgY against *C. jejuni* infection in mice

#### 3.2.1 IgY alleviated *C. jejuni*-induced clinical symptoms

**Figure 2:**
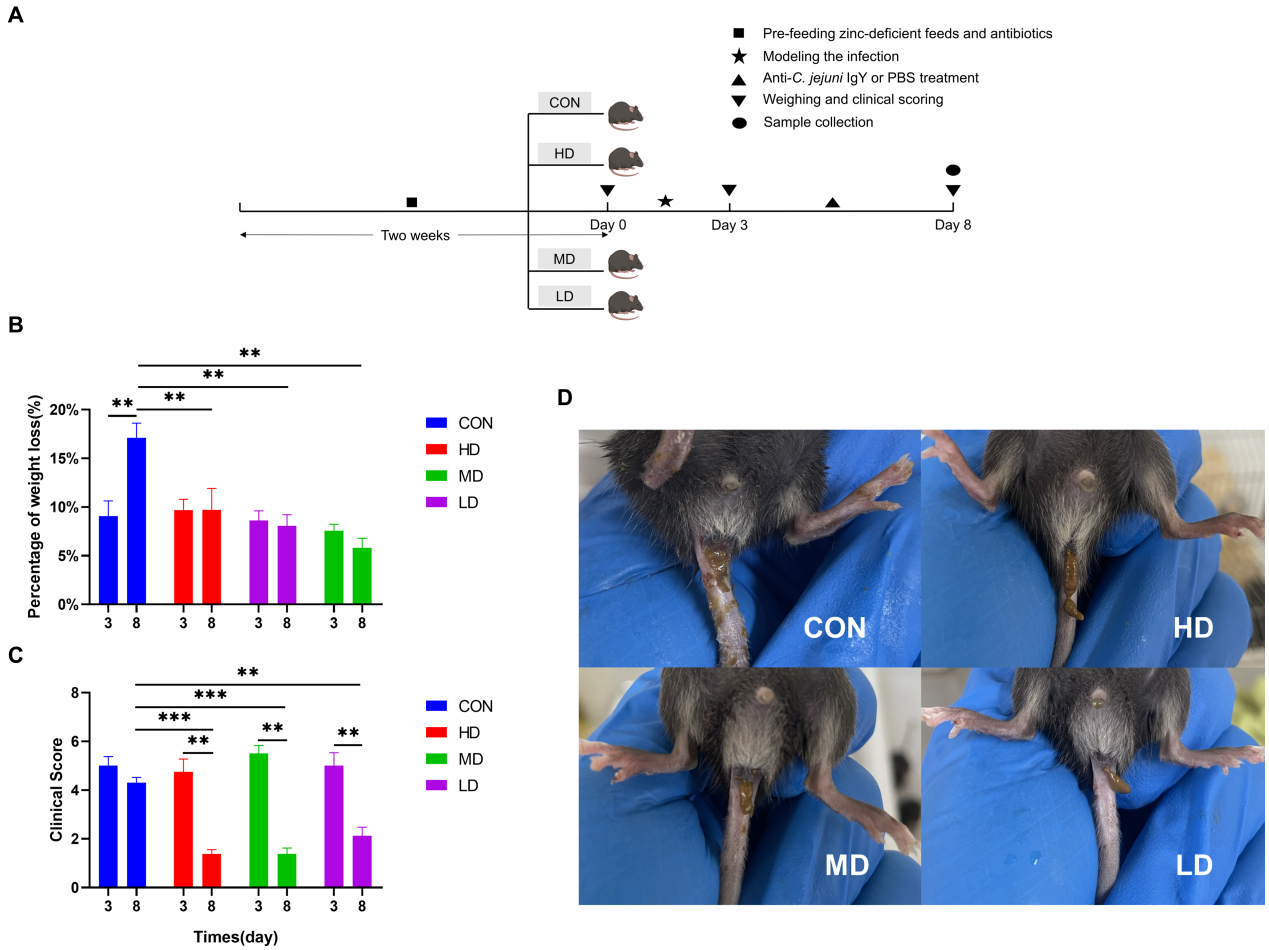
IgY blocks weight loss and improves clinical performance in mice. **(A)** Timeline of the mouse experiments. **(B)** Mouse body weights were recorded on day 0, day 3, and day 8. **(C)** Scoring of mice using a standardized clinical scoring system. **(D)** Images of mouse feces on day 8. Data are presented as mean ± SEM (*n* = 8), *p < 0.05 and **p < 0.01, and the symbol (#) represents difference tendency (*p* < 0.1).

To investigate whether oral administration of anti-*C. jejuni* IgY could alleviate the negative effects of *C. jejuni* on mice, we recorded the changes in body weight of mice on day 0 (before modeling), day 3 (after modeling), and day 8 (end of the experiment) (Figure 2A). After 3 days of gavage, the mice lost an average of 7% of their body weight. On day 8, the mice in the IgY-treated groups stopped losing body weight, while the mice in the CON group continued to lose weight. The magnitude of body weight loss in the CON group was significantly higher than that in the IgY-treated group (Figure 2B).

The standardized clinical scoring system was used to evaluate the clinical scores of the mice. On day 8, the IgY-treated groups showed significantly lower clinical scores compared to day 3 and the CON group (Figure 2C). In addition, the feces of mice treated with anti-*C. jejuni* IgY were well-formed by the 8th day (Figure 2D). These results showed that specific IgY could effectively improve the clinical symptoms caused by *C. jejuni*, with the most significant effect observed in the high-dose group.

#### 3.2.2 IgY relieved inflammation of the jejunum and colon

**Figure 3:**
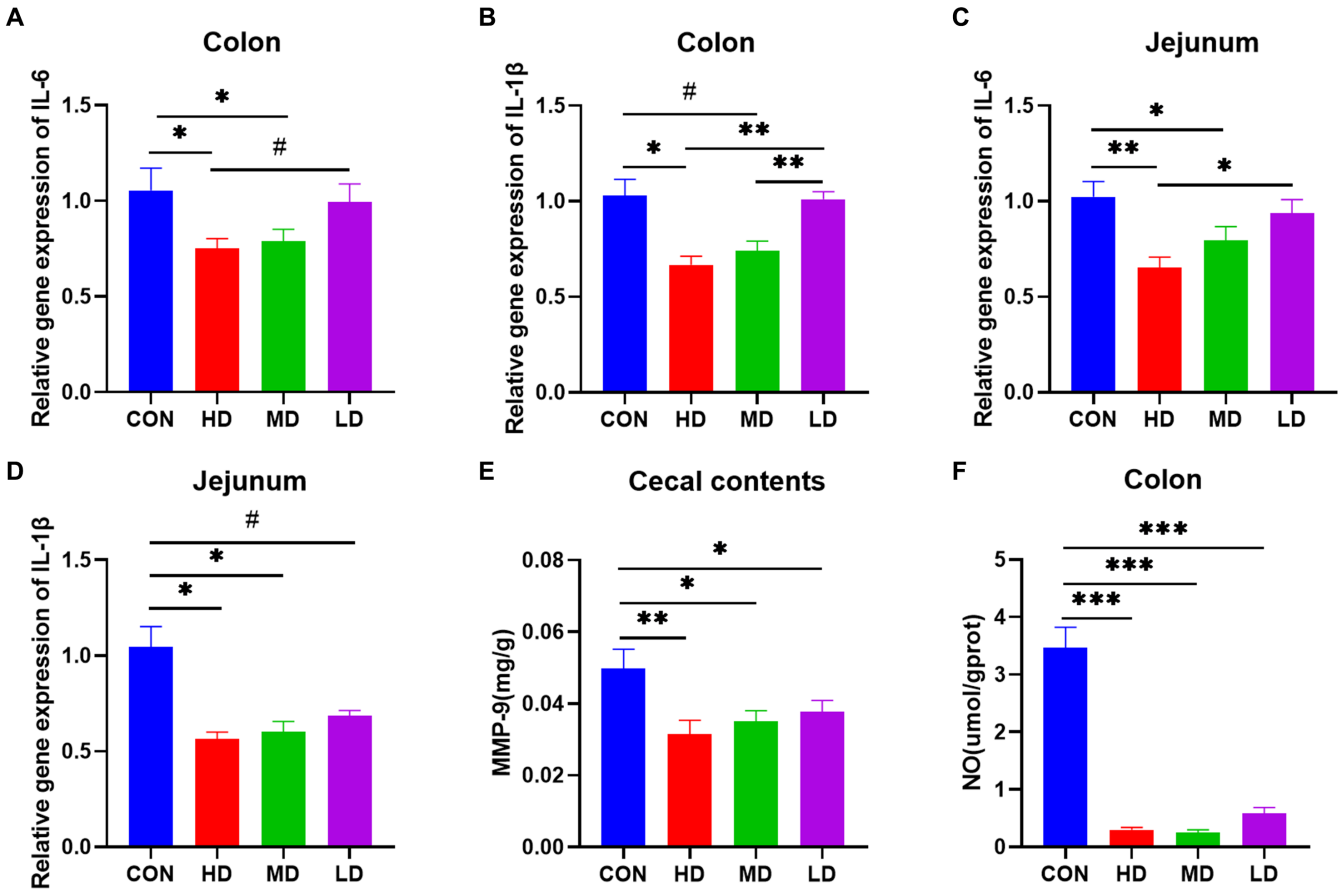
Relative mRNA expression of inflammatory genes, inflammatory markers in cecal contents, and colonic NO are decreased in the colon and jejunum of IgY-treated mice. **(A)** IL-6 mRNA expression in the colon. **(B)** IL-1β mRNA expression in the colon. **(C)** IL-6 mRNA expression in the jejunum. **(D)** IL-1β mRNA expression in the jejunum. **(E)** MMP-9 content of cecal contents. **(F)** Nitric oxide content in colon tissues. Data are presented as mean ± SEM (*n* = 8), *p < 0.05 and **p < 0.01, and the symbol (#) represents difference tendency (*p* < 0.1).

To investigate the mitigating effect of IgY on *C. jejuni*-induced intestinal inflammation, we assessed the expression of intestinal inflammatory cytokine mRNAs, the NO content of colonic tissues, and the concentration of inflammatory markers in cecal contents. The relative mRNA expression of the proinflammatory factor IL-6 in the colon of mice was significantly lower in the HD and MD groups than in the control group, and there was a trend towards lower colonic IL-6 mRNA expression in mice in the HD group compared with mice in the LD group (Figure 3A). And compared with the control group, the relative expression of IL-1β mRNA was significantly lower in the HD group and also tended to be lower in the MD group, while the relative expression of IL-1β mRNA in the colon of mice in both the HD and MD groups was highly significantly lower than that in the LD group (Figure 3B). Quantification of the relative expression of jejunal IL-6 mRNA showed similar results: the relative expression of jejunal IL-6 was significantly lower in both the HD and MD groups than in the control group, and the expression of jejunal IL-6 was significantly lower in the HD group than in the LD group (Figure 3C). For the relative expression of jejunal IL-1β mRNA, it was significantly lower in the HD and MD groups than in the control group, and there was also a trend toward a decrease in the LD group (Figure 3D). In addition, we quantified MMP-9 in cecal contents and NO in colonic tissue and found that specific IgY treatment reduced both MMP-9 and NO levels compared to controls (Figures 3E,F). Thus, IgY treatment groups, especially high-dose IgY treatment, attenuated pro-inflammatory mediator responses in the intestines of mice infected with *C. jejuni*.

#### 3.2.3 IgY inhibited *C. jejuni* colonization and increased SCFAs concentration

**Figure 4:**
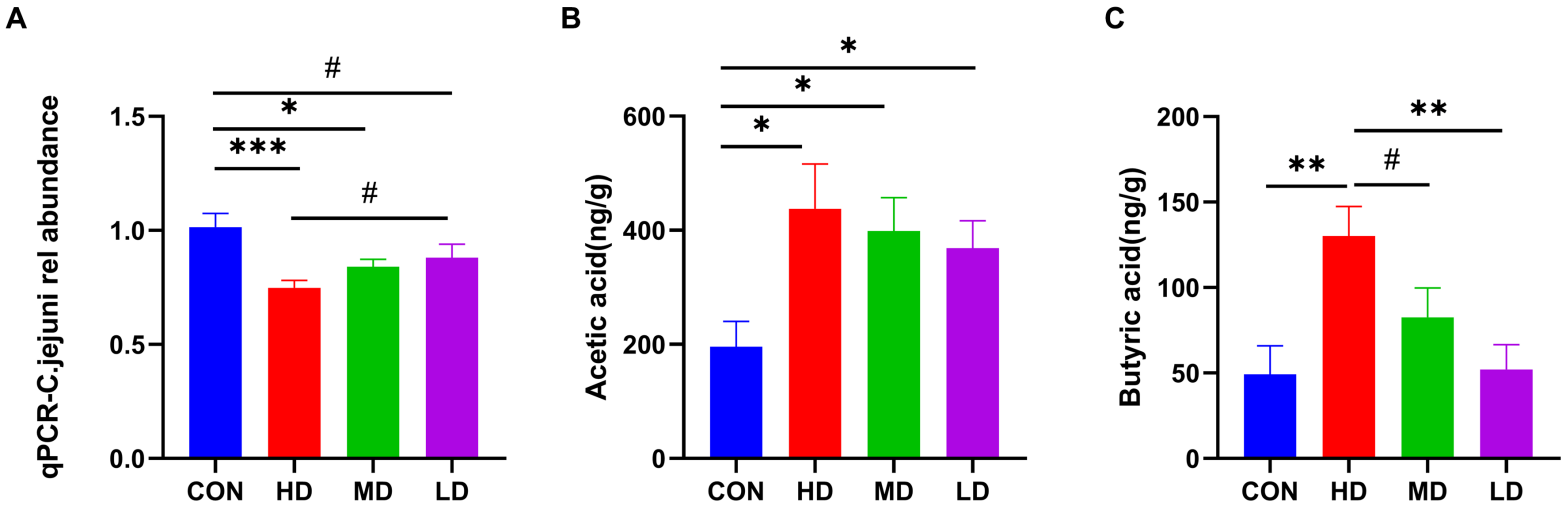
IgY reduces *C.jejuni* load and increases the fatty acid concentration in mouse cecum contents. **(A)** Relative load of *C.jejuni*. **(B)** The levels of acetic acid. **(C)** Butyric acid in mice cecum contents. Data are presented as mean ± SEM (*n* = 8), *p < 0.05, **p < 0.01, and ***p < 0.001, and the symbol (#) represents difference tendency (*p* < 0.1).

To determine whether the protective effect of IgY in mice was due to the rate of bacterial clearance, the relative number of *C. jejuni* in the cecal contents of mice at the end of the experiment was measured. The number of *C. jejuni* in the cecal contents of mice in the HD and MD groups was significantly lower than that in the control group, and there was also a trend towards less in the LD group. Furthermore, the relative number of *C. jejuni* in mouse cecal contents was reduced in a dose-dependently manner in the IgY-treated groups (Figure 4A).

In terms of SCFAs, the content of acetic acid in the cecal contents of IgY-treated mice was significantly higher than in the control group and was highest in the HD group (Figure 4B). The cecal contents of the HD group also had the highest levels of butyric acid, significantly higher than those of the control and LD groups. Additionally, there was also a tendency towards increased butyric acid content in the HD group compared to the MD group (Figure 4C). Propionic acid and valeric acid were not detected. These results suggest that high doses of anti-*C. jejuni* IgY improved the efficiency of bacterial clearance and positively influenced the production of microbial metabolites.

### 3.3 Protective effect of IgY against *C. jejuni* infection in ragdoll cats

#### 3.3.1 IgY improved the clinical presentation in ragdoll cats

**Figure 5:**
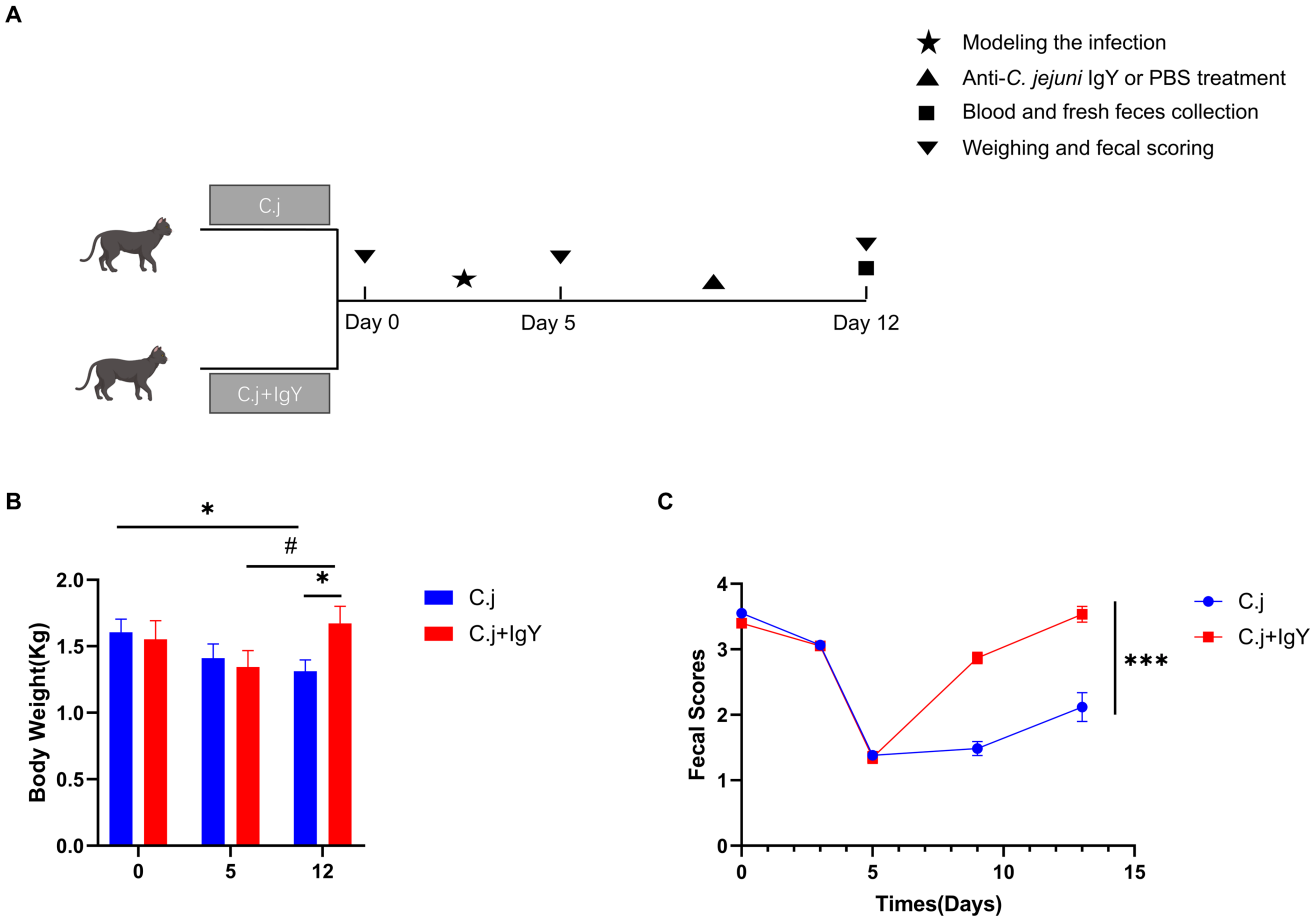
Effects of IgY on clinical signs in *C.jejuni*-infected cats. **(A)** Timeline and time points for collecting cat samples. Changes in **(B)** body weight and **(C)** fecal scores of cats in the C. j group and the C. j + IgY group. Fecal samples were scored on a scale of 1 to 5 (1 – liquid diarrhea to 5 – dry hard pellets). A score of 3.5 to 4.0 was considered ideal. Data are presented as mean ± SEM (*n* = 6), *p < 0.05, **p < 0.01, and ***p < 0.001, and the symbol (#) represents difference tendency (*p* < 0.1).

To investigate whether oral administration of anti-*C. jejuni* IgY mitigates the negative effects of *C. jejuni* on Ragdoll cats, we recorded body weights and applied a standardized fecal score system at various stages of the experimental process (Figure 5A). Throughout the experiment, Ragdoll cats in the C.j group consistently experienced weight loss, resulting in a significantly lower final body weight in comparison to their initial weight. In contrast, the Ragdoll cats in the C. j + IgY group showed a decrease after modeling (on day 5), and returned to the initial level at the end of the experiment (on day 12), which was significantly higher than that of the C. j group (Figure 5B). Regarding fecal scores, both groups of Ragdoll cats experienced vomiting and diarrhea on day 5. At the end of the experiment, Ragdoll cats in the IgY group exhibited well-formed stools, whereas the cats in the C. j group continued to experience soft stools and diarrhea (Figure 5C).

#### 3.3.2 Effect of IgY on serum biochemical and inflammation-related parameters

**Figure 6:**
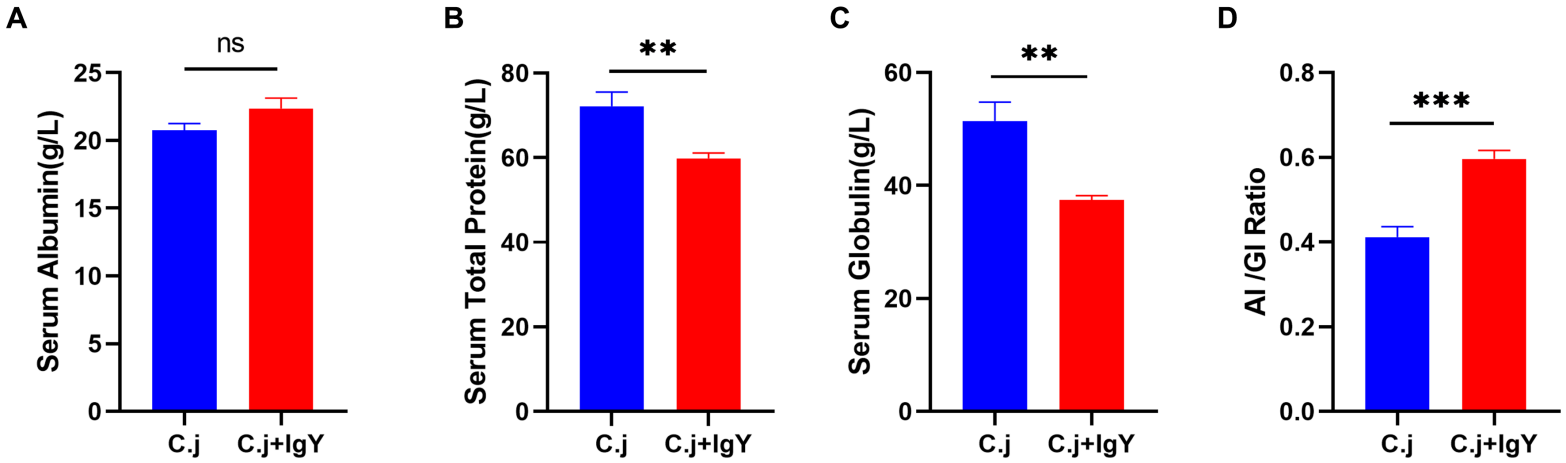
Effect of anti-*C. jejuni* IgY on serum biochemical parameters in cats. The levels of **(A)** Serum albumin, **(B)** Serum total protein, **(C)** Serum globulin, and **(D)** Al/Gl Ratio (Albumin to globulin ratio). Data are presented as mean ± SEM (*n* = 6), *p < 0.05, **p < 0.01, and ***p < 0.001, ns, not significant, and the symbol (#) represents the difference tendency (*p* < 0.1).

To assess the effect of IgY on liver function in *C. jejuni*-infected Ragdoll cats, the total protein, albumin, globulin, and the AL/GI ratio in serum were determined. These parameters are indicative of liver function and immune response. There was no significant difference in serum albumin levels between the two groups (Figure 6A), while serum total protein (Figure 6B) and globulin (Figure 6C) were significantly higher in the C. j group than in the C. j + IgY group. The serum AI/GI ratio was significantly lower in the C. j group than in the C. j + IgY group (Figure 6D).

**Figure 7:**
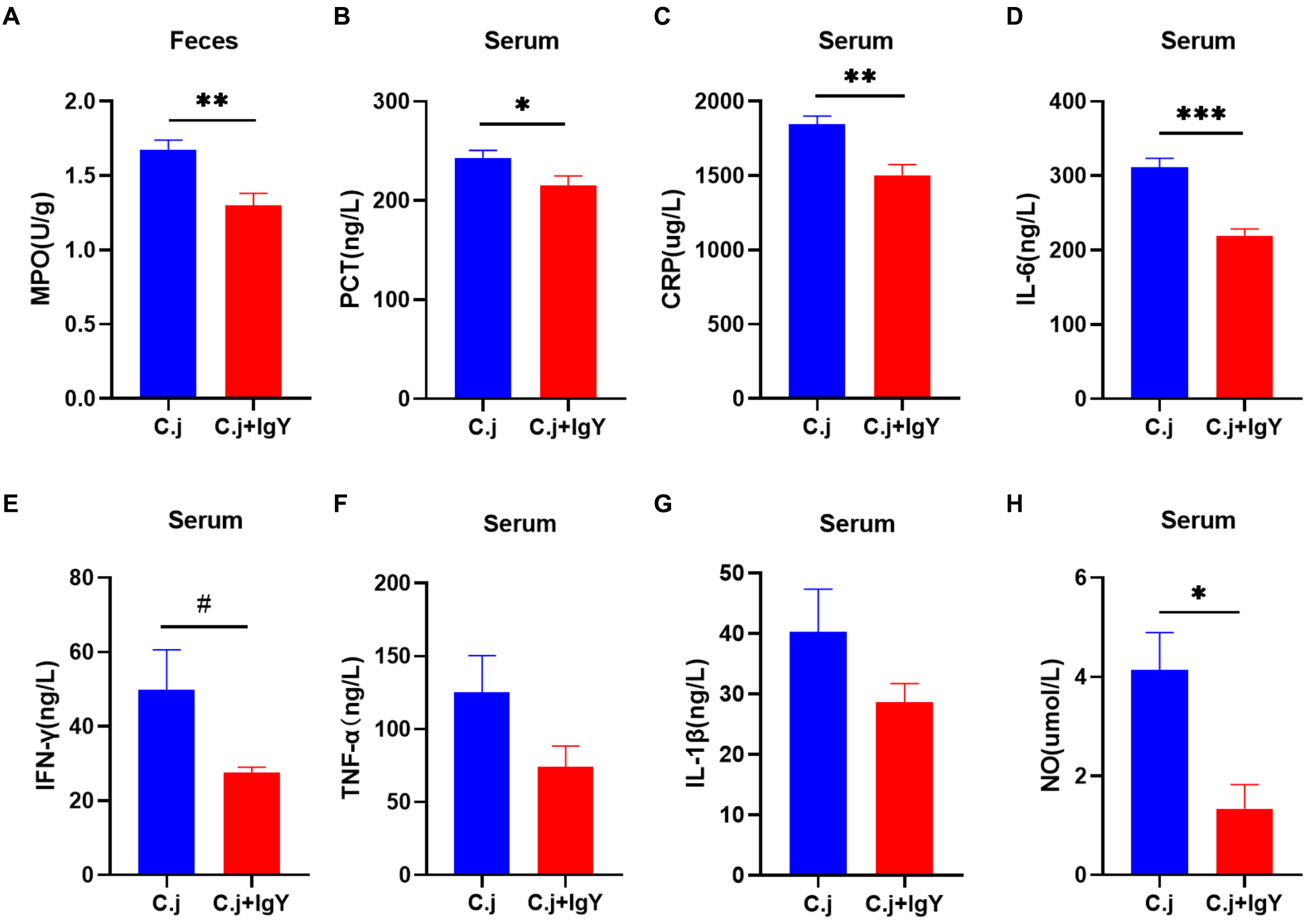
Anti-*C. jejuni* IgY attenuated inflammation caused by *C. jejuni* infection. The levels of **(A)** MPO, **(B)** PCT, **(C)** CRP, **(D)** IL-6, **(E)** IFN-γ, **(F)** TNF-α, **(G)** IL-1β, and **(H)** NO in serum. Data are presented as mean ± SEM (*n* = 6), *p < 0.05, **p < 0.01, and ***p < 0.001, and the symbol (#) represents difference tendency (*p* < 0.1).

*C. jejuni* causes hyper-immunity and elicits a systemic inflammatory response. We then investigated the effect of anti-*C. jejuni* IgY on systemic inflammation in cats. The fecal marker of inflammation, MPO, was significantly reduced after specific IgY treatment (Figure 7A). Serum levels of PCT and CRP, two markers of bacterial infection, were significantly lower in cats in the C.j + IgY group (Figures 7B,C). In addition, the pro-inflammatory cytokine IL-6 (Figure 7D) was significantly lower in the serum of cats in the C.j + IgY group than in the C.j group, and IFN-γ (Figure 7E) also tended to be lower. This was accompanied by lower levels of TNF-α and IL-1β (Figures 7F,G). It was also observed that serum NO levels were significantly decreased after specific IgY treatment (Figure 7H). These results suggest that IgY treatment can attenuate the systemic inflammatory response.

#### 3.3.3 IgY increased SCFAs levels in ragdoll cats

**Figure 8:**
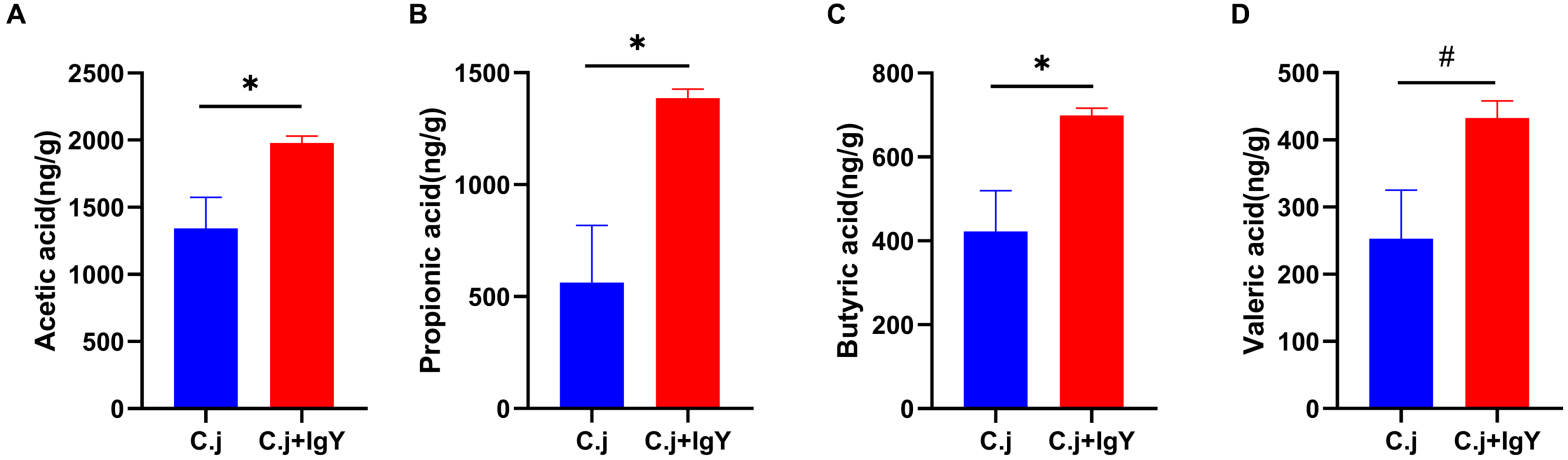
Effect of Anti-*C. jejuni* IgY on SCFAs in cat feces. The levels of **(A)** Acetic acid, **(B)** Propionic acid, **(C)** Butyric acid, and **(D)** Valeric acid in cat feces. Data are presented as mean ± SEM (*n* = 6), *p < 0.05, **p < 0.01, and ***p < 0.001, and the symbol (#) represents difference tendency (*p* < 0.1).

SCFAs are an important source of energy for colonic epithelial cells. Measurement of the content of SCFAs in the fresh feces of Ragdoll cats showed that the levels of acetic acid (Figure 8A), propionic acid (Figure 8B), and butyric acid (Figure 8C) were all significantly higher in the C. j + IgY group. The level of valeric acid (Figure 8D) also tended to be elevated.

#### 3.3.4 Effect of anti-*C. jejuni* IgY on the untargeted metabolomics of cat feces

**Figure 9:**
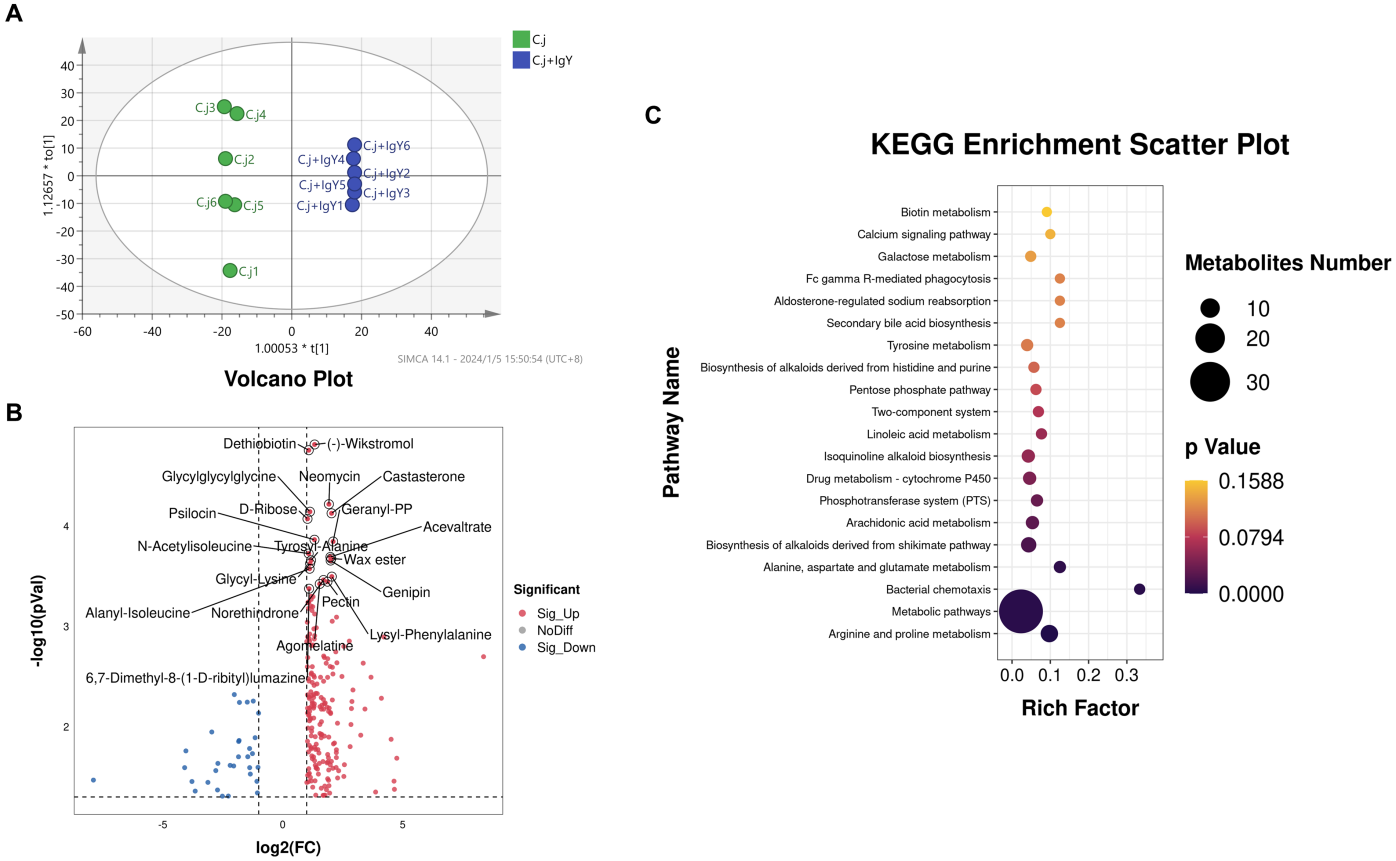
Effect of anti-*C.jejuni* IgY on the untargeted metabolomics of cat feces. **(A)** Score plots for the orthogonal partial least squares discriminant analysis (OPLS-DA) model between the two groups. **(B)** Volcano plot of differential metabolites in the feces (C. j + IgY vs. C. j). **(C)** Bubble diagram of metabolic pathway analysis for two groups of differential metabolites.

The OPLS-DA score plots showed the separation between the C. j and C. j + IgY groups indicating significant differences in fecal metabolites between the two groups (Figure 9A). Further analysis identified 233 differential metabolites, of which 202 were upregulated and 31 were downregulated (Figure 9B). The KEGG enrichment analysis revealed that the differential metabolites were primarily enriched in arginine and proline metabolism, alanine, aspartate and glutamate metabolism, and bacterial chemotaxis (Figure 9C). These amino acids are essential for the metabolism of *C. jejuni*, and bacterial chemotaxis is associated with its colonization and proliferation.

## 4 Discussion

Domestic cats are important hosts for *C. jejuni*, which can lead to subclinical infections and even the development of enteritis (Acke, 2018). After infection, *C. jejuni* rapidly colonizes the intestine, translocates through the intestinal mucus, and can invade tissues, causing inflammation (Backert et al., 2013) and some highly virulent strains can cause severe diarrhea with bloody stools (Lee et al., 2003).

To evaluate the effectiveness of anti-*C. jejuni* IgY in alleviating symptoms caused by *C. jejuni* infection, we established mouse and cat infection models. The infected animals showed obvious signs of infection such as persistent diarrhea, weight loss, and frequent vomiting in cats, suggesting that our model is suitable for assessing the effectiveness of IgY against *C. jejuni* infection. Anti-*C. jejuni* IgY effectively alleviated clinical signs of *C. jejuni* infection, including weight gain and well-formed feces.

*Campylobacter jejuni* infection triggers the immune system, leading to an increase in pro-inflammatory factors such as IL-1β, IL-6, and IFN-γ. Overexpression of these cytokines can be detrimental to health (Haitao et al., 2018). *C. jejuni* infection upregulates the expression of IL-6 and IL-1β in the colon and jejunum of mice, as well as the levels of IL-6 and IFN-γ in the serum of cats. IL-1β induces signal transduction and kinase activation, leading to substrate phosphorylation and enhanced DNA-binding activity of the nuclear factor, which in turn induces the expression of MMP-9 (Shaista et al., 2002). Schnbeck et al. (1998) found that MMP-9 can cleave IL-1 at the site of action of the IL-1β converting enzyme, resulting in the conversion of the inactive precursor pIL-1β (33 kD) to the active IL-1β (17/28 kD). The positive feedback regulation of MMP-9 with IL-1β exacerbates the inflammatory response. Our study found that IgY treatment reduced the levels of these inflammatory factors to varying degrees. CRP and PCT are markers of bacterial infection (Liliana et al., 2004). CRP is an acute response protein produced by the liver that can identify bacterial or viral infections (Coster and Wasserman, 2020). In viral infections, the level of CRP is typically not elevated. However, in bacterial infections, it begin to rise 4–6 h after infection and decreases once the infection is under control. PCT is another marker of bacterial infection, which is normally confined mainly to the parathyroid glands has low serum levels, and responds more rapidly to bacterial infections, especially gram-negative bacterial infections (Bassetti et al., 2018). Our studies showed that anti-*C. jejuni* IgY treatment was effective in reducing serum levels of CRP and PCT in cats. MPO is a reliable biomarker of inflammatory bowel disease (Hansberry et al., 2017). *C. jejuni* infection leads to intestinal inflammation, causing neutrophils to recruit to the intestine and release MPO, and fecal MPO levels are positively correlated with the degree of intestinal inflammation (Krawisz et al., 1984). Cats treated with anti-*C. jejuni* IgY showed a significant decrease in fecal myeloperoxidase levels, accompanied by a decrease in serum nitric oxide levels. These data suggest that treatment with specific IgY can effectively inhibit the inflammatory response induced by *C. jejuni*.

Gut microbiota-derived metabolites are crucial for the integrity of the intestinal barrier. SCFAs produced by intestinal microorganisms through carbohydrate fermentation play an important role in various physiological processes in the body (Koh et al., 2016). *C. jejuni* colonizes the gut and disrupts the host’s normal intestinal flora, creating competition for nutrients and space between foreign pathogens and the host’s intestinal commensals. This effect was particularly evident in the antibiotic-scavenged intestines of the mice. Our study found that the relative load of *C. jejuni* decreased significantly in the number of *C. jejuni* in the HD and MD groups, and the concentration of SCFAs increased with the use of anti-*C. jejuni* IgY. This suggests that anti-*C. jejuni* IgY enhances the excretion of *C. jejuni* and limits its colonization of the intestine, thereby improving the metabolism of intestinal microorganisms.

To further investigate how IgY confers protection, we conducted an untargeted metabolomics study of Ragdoll cat feces. We identified 233 differential metabolites, of which 202 were up-regulated and 31 were down-regulated. Mapping these metabolites to the KEGG metabolic network revealed that IgY mainly affects amino acid metabolism and bacterial chemotaxis. *C. jejuni* cannot use sugar as a carbon source because it lacks the glycolytic enzyme phosphofructokinase (Parkhill et al., 2000). However, it can utilize aspartic acid, proline, and glutamic acid, as demonstrated in early *in vitro* experiments (Leach et al., 1997; Edward Guccione et al., 2010; Leon-Kempis et al., 2010), Parsons et al. quantified the chicken gut and found high levels of amino acids on which *C. jejuni* depends (Parsons et al., 1983). The acquisition of nutrients is a key factor in bacterial colonization and pathogenesis. *C. jejuni* can detect the concentration of certain components around the intestinal mucosa through chemotactic sensors, the combination of motility and chemotactic cascade responses facilitates *C. jejuni* colonization at suitable sites for growth (Korolik, 2019). Changes in these amino acid-related pathways and the effect of specific IgY on bacterial chemotaxis may be important in the clearance of *C. jejuni* from the gut.

Our study showed that specific IgY had potent inhibitory activity *in vitro* and effectively ameliorated the inflammatory response and intestinal structural damage induced by *C. jejuni*. Anti-*C. jejuni* IgY reduced the bacterial load of cecal contents and gut colonization through mechanisms that may involve amino acid metabolism and bacterial chemotaxis. The results suggest that anti-*C. jejuni* IgY can help protect gut health and modulate inflammatory responses. In conclusion, anti-*C. jejuni* IgY could potentially offer passive immune protection against pathogenic bacterial infections.

The study also had several limitations. Although clinical signs such as diarrhea and weight loss were observed in both the mouse and Ragdoll cat infection models, significant bloody stools were not observed. This may be due to the low virulence of the isolated *C. jejuni*. It is unclear whether this antibody has similar effects on different subspecies or serotypes of *C. jejuni*, and further in-depth studies are necessary.

## Data availability statement

The raw data supporting the conclusions of this article will be made available by the authors, without undue reservation.

## Ethics statement

The animal study was reviewed and approved by the Animal Care and Use Committee; the Laboratory Animal Center at the South China Agricultural University.

## Author contributions

ZL: Data curation, Methodology, Software, Writing – original draft, Writing – review & editing. JY: Conceptualization, Investigation, Methodology, Visualization, Writing – review & editing. ZB: Conceptualization, Data curation, Investigation, Supervision, Writing – review & editing. JZ: Data curation, Investigation, Software, Supervision, Writing – review & editing. YL: Data curation, Investigation, Validation, Writing – review & editing. JD: Writing – review & editing, Conceptualization, Funding acquisition. BD: Conceptualization, Supervision, Writing – review & editing. SH: Conceptualization, Funding acquisition, Methodology, Project administration, Resources, Supervision, Writing – review & editing, Data curation.

## Footnotes

^1^ https://www.36kr.com/p/2155618644196871
