# Supplementary material for: Protective effect of chicken yolk antibody Y against Campylobacter jejuni induced diarrhea in cats
Source: Front Microbiol. 2024 Apr 9;15:1378029. doi: 10.3389/fmicb.2024.1378029 (PMC11037399; doi:10.3389/fmicb.2024.1378029)
Supplement: Supplementary file 1 [file Table_1.DOCX]

Supplementary Table 1. Primer sequences for qPCR

| Gene name | Forward sequence | Reverse sequence |
| --- | --- | --- |
| IL-6 | ATAGTCCTTCCTACCCCAATTTCC | CTGACCACAGTGAGGAATGTCCAC |
| IL-1β | GAAATGCCACCTTTTGACAGTG | TGGATGCTCTCATCAGGACAG |
| β-actin | CTGTCCCTGTATGCCTCTG | ATGTCACGCACGATTTCC |
| C.jejuni | TCCATCATATCTTGGGCGCT | AATTTGCTTTGAAAGCATTT |
| Universal bacterial | GTGSTGCAYGGYYGTCGTCA | ACGTCRTCCMCNCCTTCCTC |
